# From Genes to Environment: Elucidating Pancreatic Carcinogenesis Through Genetically Engineered and Risk Factor-Integrated Mouse Models

**DOI:** 10.3390/cancers17101676

**Published:** 2025-05-15

**Authors:** Bin Yan, Anne-Kristin Fritsche, Erik Haußner, Tanvi Vikrant Inamdar, Helmut Laumen, Michael Boettcher, Martin Gericke, Patrick Michl, Jonas Rosendahl

**Affiliations:** 1 Department of Internal Medicine IV, Heidelberg University Hospital, 69120 Heidelberg, Germany; bin.yan@med.uni-heidelberg.de; 2 Institute of Anatomy and Cell Biology, Martin-Luther-University Halle-Wittenberg, 06120 Halle (Saale), Germany; anne-kristin.fritsche@medizin.uni-leipzig.de; 3 Institute of Anatomy, Leipzig University, 04103 Leipzig, Germany; martin.gericke@medizin.uni-leipzig.de; 4 Institute of Molecular Medicine, Section for Molecular Medicine of Signal Transduction, Faculty of Medicine, Martin-Luther-University Halle-Wittenberg, 06120 Halle (Saale), Germany; erik.haussner@uk-halle.de (E.H.); michael.boettcher@medizin.uni-halle.de (M.B.); 5 Department of Internal Medicine I, Martin-Luther-University Halle-Wittenberg, 06120 Halle (Saale), Germany; tanvivikrant.inamdar@uk-halle.de (T.V.I.); helmut.laumen@medizin.uni-halle.de (H.L.)

**Keywords:** genetically engineered mouse models, pancreatic cancer, carcinogenesis, risk factors, pancreatitis, obesity, diabetes

## Abstract

Pancreatic cancer is characterized by late diagnosis, therapy resistance, and poor prognosis, necessitating the exploration of early carcinogenesis and prevention methods. Preclinical mouse models have evolved from cell line-based to human tumor tissue- or organoid-derived xenografts, now to humanized mouse models and genetically engineered mouse models (GEMMs). GEMMs, primarily driven by oncogenic Kras mutations and tumor suppressor gene alterations, offer a realistic platform for investigating pancreatic cancer initiation, progression, and metastasis. The incorporation of inducible somatic mutations and CRISPR-Cas9 screening methods has expanded their utility. To better recapitulate tumor initiation triggered by inflammatory cues, common pancreatic risk factors are being integrated into model designs. This approach aims to decipher the role of environmental factors as secondary or parallel triggers of tumor initiation alongside oncogenic burdens. Emerging models exploring pancreatitis, obesity, diabetes, and other risk factors offer significant translational potential. This review describes current mouse models for studying pancreatic carcinogenesis, their combination with inflammatory factors, and their utility in evaluating pathogenesis, providing guidance for selecting the most suitable models for pancreatic cancer research.

## Simple Summary

Pancreatic cancer remains one of the deadliest malignancies. This review summarizes preclinical mouse models in early pancreatic cancer research, focusing on genetically engineered models ranging from transgenic to inducible gene editing approaches. These models offer diverse platforms for deciphering carcinogenesis mechanisms and testing therapeutic strategies. We emphasize their applications in investigating environmental risk factors, aiming to provide researchers with a better understanding of their utility and limitations in translational pancreatic cancer research.

## 1. Introduction

Pancreatic cancer remains a tremendous challenge in oncology, ranking the third leading cause of cancer deaths [1]. Despite modest improvements in recent years, the 5-year relative survival rate for pancreatic cancer stands at 13%. Pancreatic ductal adenocarcinoma (PDAC), the most prevalent type of pancreatic cancer, is characterized by its aggressive nature, lack of early detection methods, and an immunosuppressive tumor microenvironment [2,3]. These factors contribute to a narrow therapeutic window, underscoring the critical need for research focused on risk factors and early carcinogenesis. Understanding the key genetic and environmental risk factors that drive pancreatic cancer development is essential for improving early detection strategies, prevention approaches, and treatment outcomes.

**Figure 1:**
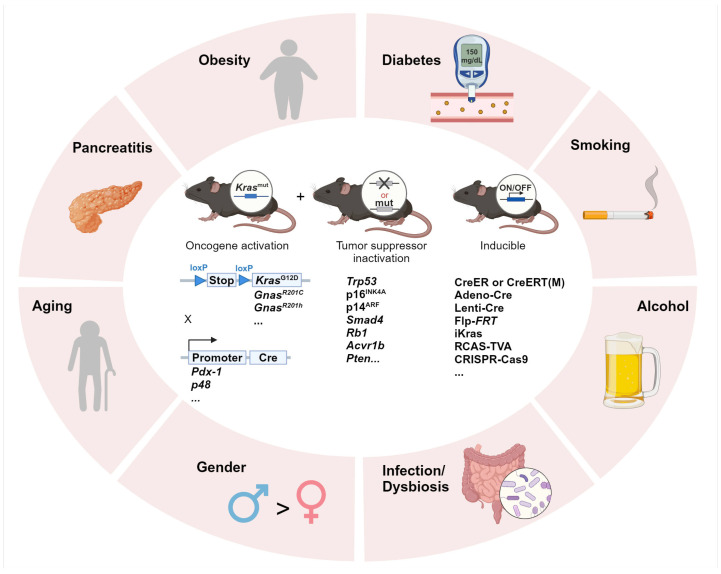
Integrating environmental risk factors into pancreatic carcinogenesis research using GEMMs. Summary of the commonly investigated environmental risk factors in PDAC research and GEMMs of transgenic or inducible oncogene activation and/or tumor suppressor gene inactivation. Adeno-Cre: adenoviral-Cre; Lenti-Cre: lentiviral-Cre; Flp-FRT: flippase-FRT; iKras: doxycycline-inducible Kras; RCAS: replication-competent avian sarcoma-leukosis virus long terminal repeat with splice acceptor; TVA: tumor virus A; CRISPR: clustered regularly interspaced short palindromic repeats; Cas9: CRISPR-associated 9.

Epidemiological studies have identified several well-established risk factors for pancreatic cancer, including both modifiable lifestyle/environmental factors and non-modifiable factors such as age and genetic predisposition [4] (Figure 1). Among the modifiable factors, smoking is one of the most significant with the highest risk observed in heavy smokers compared to non-smokers, and the elevated risk persists for at least 10 years after cessation [5]. Alcohol consumption is also associated with increased risk, either through direct genotoxic effects on the pancreas or indirectly via the induction of pancreatitis [6]. Pancreatitis, particularly chronic pancreatitis, significantly elevates pancreatic cancer risk, with acute pancreatitis also contributing to short-term risk within the first three years after diagnosis [7,8]. Furthermore, obesity is another major modifiable risk factor, often associated with poor prognosis and treatment outcomes in pancreatic cancer patients [9,10]. Obesity-induced insulin resistance can lead to type 2 diabetes, which—along with non-obese diabetes—is a recognized risk factor. Infections with pathogens such as Hepatitis B and C viruses and Helicobacter pylori have also been implicated in pancreatic cancer development, suggesting a role for microbiome alterations in disease progression [11,12].

In addition to modifiable factors, non-modifiable contributors also play a significant role. Aging is strongly associated with pancreatic cancer incidence, with most PDAC diagnoses occurring between 60 and 80 years of age [13]. The accumulation of oncogenic mutations over time, along with inherited genetic predispositions, further increases susceptibility.

Understanding how these risk factors influence the molecular progression of pancreatic carcinogenesis is critical. Pancreatic carcinogenesis typically progresses through a series of sequential steps, often beginning with acinar-to-ductal metaplasia (ADM), which is initially reversible [14]. However, with persistent inflammation or injury, as induced by the risk factors described above, ADM can progress to premalignant pancreatic intraepithelial neoplasias (PanINs). These PanINs are graded from low-grade (PanIN-1 and PanIN-2) to high-grade (PanIN-3) lesions, which can ultimately develop into invasive PDAC [15]. This progression is induced or accompanied by the accumulation of genetic alterations, including activating mutations in the *Kras* oncogene (in >90% of PDAC cases) and inactivation of tumor suppressor genes such as *CDKN2A*, *TP53*, and *SMAD4* [16]. Alternative precursor lesions, including intraductal papillary mucinous neoplasms (IPMNs) and mucinous cystic neoplasms (MCNs), represent additional routes to PDAC [17].

**Figure 2:**
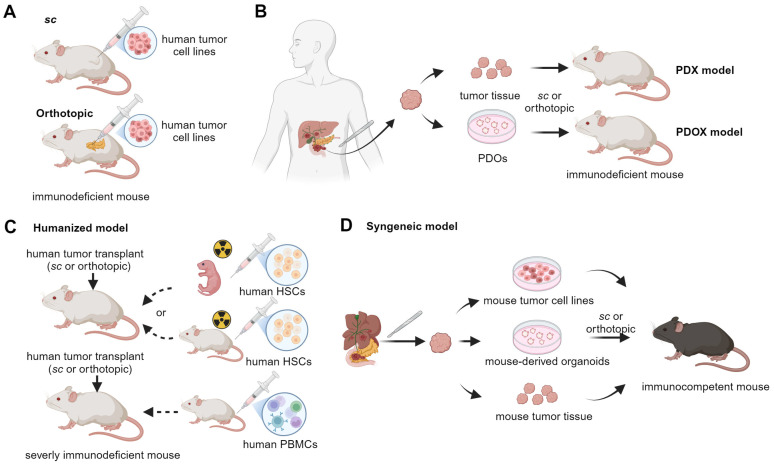
Transplantation-based mouse models studying PDAC. (**A**) Subcutaneous (*sc*) and orthotopic models are generated in immunodeficient mice through the injection of tumor cells under the skin and into the pancreas, respectively. (**B**) Immunocompromised mice are transplanted either subcutaneously or orthotopically with human tumor tissues for PDX (patient-derived xenografts) models or with PDOs (patient-derived organoids) for PDOX (patient-derived organoid xenografts) models. (**C**) Humanized mouse models are developed by first injecting peripheral blood mononuclear cells (PBMCs) into an adult severely immunodeficient mouse or by first engrafting hematopoietic stem cells (HSCs) into irradiated neonatal or adult severely immunodeficient mice. Then, the mice are transplanted with human tumor tissues (cell line, PDX, PDOX, etc.). (**D**) Syngeneic mouse models are generated by injecting mouse tumor materials subcutaneously or orthotopically. These materials include cell lines, PDX (patient-derived xenografts), and PDOX (patient-derived orthotopic xenografts) developed from mouse tumors.

While existing preclinical mouse models have significantly advanced our understanding of pancreatic tumor biology and therapy, many fail to accurately replicate the early events of tumorigenesis or to include risk factors [18]. Traditional xenograft models using established cell lines typically represent advanced disease states and lack the immune components [19] (Figure 2A). Patient-derived xenografts (PDXs) and patient-derived organoid xenografts (PDOXs) have further improved the retention of parental tumor features such as heterogeneity, but they also rely on immunodeficient hosts and fail to replicate PDAC development in the context of native inflammatory and immune environment [20] (Figure 2B). To better capture tumor–immune dynamics, humanized mouse models, syngeneic models, and GEMMs have been developed. Humanized mouse models, lacking B, T, and NK cell activities, allow the engraftment of human tumor tissue and immune cells, thereby restoring some human-specific immune–tumor interactions while retaining interpatient tumor heterogeneity (Figure 2C). These models, however, exhibit mismatched stromal environments and do not recapitulate de novo tumor initiation [21,22]. Syngeneic models, involving the implantation of Panc02 [23] or *Kras^G12D^*; *Trp53^R172H^*; *Pdx-1-Cre* (KPC)-derived cell lines into immunocompetent mice, allow for the study of tumor-immune interactions in genetically matched hosts [24] (Figure 2D). Nevertheless, these models remain implantation-based and genetically uniform. In contrast, GEMMs support spontaneous tumor initiation and progression, capturing dynamic tumor–stroma–immune interactions. Yet, they are typically based on engineered mutations and may not incorporate the full spectrum of environmental, metabolic, or inflammatory factors that drive human disease. Constructing GEMMs can be labor-intensive and costly, requiring specialized equipment and expertise, and the success rate of generating GEMMs can also vary depending on the specific genetic alterations and the strain of mice used [25]. Despite these challenges, GEMMs remain a cornerstone of PDAC research, offering valuable insights into the molecular mechanisms of PDAC, particularly when used in conjunction with relevant risk factors.

This review aims to provide an overview of the most widely used and promising GEMMs in pancreatic carcinogenesis, with a further emphasis on their applications in studying modifiable risk factors such as pancreatitis, obesity, and diabetes, which represent major modifiable risk factors for human PDAC. Additionally, we will explore the cutting-edge engineering methods including genome-wide and targeted CRISPR screening methods that have expanded the utility of animal models, enabling more precise and comprehensive studies of pancreatic cancer biology.

## 2. GEMMs

GEMMs have been instrumental in elucidating the mechanisms of PDAC initiation and progression. While comprehensive overviews of existing GEMMs are available elsewhere [26,27], here we briefly highlight the most relevant models that form the basis for studying genetic changes in PDAC, especially those that can later be used to explore how risk factors affect the disease.

Pioneering animal studies first utilized the elastase (EL) promoter controlled SV40 T-antigen [28], c-H-*ras* [29], Myc [30], and TGF-α [31] to study the roles of these genes during early cancer development. Among these, only TGF-α activation can induce ADM followed by fibrosis and eventually advanced PDAC, while the other genes primarily affect acinar cells. Moreover, these mice did not develop PDAC through well-defined PanIN. The discovery that *KRAS* mutations—especially the G12D isoform—occur in over 90% of PanIN-1 lesions [32] and increase with disease stage led to the development of the Lox-Stop-Lox (LSL)-*Kras^G12D/+^* model (KC mice), controlled by Cre recombinase under pancreas-specific promoter *Pdx-1* or *p48* (*Ptf1a*) [33]. This model faithfully recapitulates human PanIN progression with the PanIN-1 lesions appearing by 8 weeks of age but rarely advances to invasive PDAC without additional genetic events or environmental stimulus.

Concomitant expression of heterozygous *Trp53^R172H^* and *Kras^G12D^* in the mouse pancreas, known as the KPC model, induce invasive and widely metastatic PDAC [34], while mice with homozygous *Trp53* mutations (KPPC) develop more aggressive tumors with faster growth and shorter survival [35]. In contrast, mice with *Trp53* depletion and *Kras^G12D/+^* develop similar PanIN lesions and tumor stages as KPC mice but show significantly less metastasis [36]. Other combinations, such as *Rb1* loss [37] or *Ink4a/Arf* deletion [38] with *Kras^G12D^*, also result in aggressive tumorigenesis due to the disruption of cell cycle regulation pathways.

Notably, concomitant deletion of *Smad4* with mutant *Kras^G12D^* promotes tumor progression via IPMN/MCN lesions [39]. This SMAD4-dependent phenotype may be influenced by TGF-β superfamily ligands such as activin, as loss of activin receptor 1B (ACVR1B) accelerates IPMN-to-PDAC progression in the context of oncogenic *Kras* [40]. Interestingly, inactivation of TGFβR2, an upstream receptor of SMAD4, also enhances PanIN and PDAC development in a SMAD4-independent manner [41]. While TGF-β generally acts as a tumor suppressor, it may shift to a tumor-promoting role during later stages through interaction with the stroma, underscoring the complex and context-dependent role of TGF-β signaling in PDAC [42]. Similarly, *Gnas* gain-of-function mutation (R201C [43] or R201H [44]) induces low-grade IPMN that, when combined with oncogenic *Kras*, results in accelerated progression to invasive PDAC.

GEMMs also serve as valuable platforms for evaluating therapeutic strategies targeting the tumor microenvironment in PDAC. For example, stromal depletion through sonic hedgehog (SHH) loss [45] or αSMA+ myofibroblast ablation [46] enhances tumor aggressiveness in the background of oncogenic *Kras*, challenging classical views of the stroma as predominantly tumor-promoting [47]. Moreover, GEMMs enable preclinical testing of immunotherapies, such as checkpoint inhibitors (α-CTLA-4 and α-PD-L1) [48], as well as combination therapies (e.g., gemcitabine plus VEGFR/EGFR inhibitors [26]), which have shown encouraging outcomes. These models are thus critical for translating mechanistic findings into therapeutic advances, as reviewed in depth by Gopinathan et al. [49].

Although GEMMs may not fully capture the high mutation burden and abundant neoantigens seen in human PDAC, they offer unique advantages in modeling specific genetic alterations and their functional consequences. Importantly, these models serve as the foundation for incorporating various environmental and physiological risk factors, which will be discussed in detail in the following sections.

## 3. Inducible Somatic Gene Editing Models

To more closely mimic the somatic mutations that temporally occur in human pancreatic cancer, different inducible somatic mutation models prompt special tools to control gene expression. One of the most commonly applied tools is the inducible Cre system. The CreER system or the advanced version CreERT(M) utilizes a fusion of Cre recombinase with a modified estrogen receptor that allows tamoxifen-inducible Cre activity. Lee et al. used *Kras^LSL-G12D^*; *Trp53^flox/flox^*; *Sox9^CreERTM^* and *Kras^LSL-G12D^*; *Trp53^flox/flox^*; *Ptf1a^CreERTM^* to manipulate the gene expressions of adult mice in ductal and acinar cells, respectively, and found that ductal cells are more primed readily to form carcinoma upon oncogenic *Kras* and *Trp53* deletion, while acinar cells require longer period and develop from widespread low-grade PanINs to PDAC [50]. To model PDAC-induced cachexia, *Kras^G12D/+^*; *Ptf1a^ER-Cre/+^*; *Pten^flox/flox^* (KPP) mice were generated with controlled activation of oncogenic *Kras* and *Pten* depletion post-natally. The mice developed moderate PDAC and also exhibited a progressive wasting phenotype that closely mimics human PDAC-associated cachexia [51]. The Cre recombinase can also be delivered by retrograde pancreatic ductal injection of both adenoviral-Cre (Adeno-Cre) and lentiviral-Cre (Lenti-Cre) vectors to activate the LSL-*Kras*^G12D^ allele in the pancreas [52].

An alternative time- and host-specific recombination system that can be used alongside or instead of Cre-lox is flippase-*FRT* (Flp-*FRT*) [53]. In the *FSF-Kras^G12D/++^*; *FSF-R26^CAG−CreERT2/++^*; *Trp53^flox/flox^*; *Pdx1-Flp* mice, under the control of Pdx1-Flip, oncogenic *Kras* and CreERTM can be induced in the pancreas. After Tamoxifen administration, Cre functions to deplete *Trp53* by the Cre-loxP system. This dual-recombinase system can also be applied to study the tumor microenvironment. For example, in *FSF-Kras^G12D/+^*; *Trp53^frt/frt^*; *Pdx1-Flp; αSMA-Cre; Col1a1^flox/flox^* mice, oncogenic *Kras* is controlled by Flp-*FRT* in the pancreas and the Col1a1 is depleted in the myofibroblasts, allowing for more sophisticated spatial genetic manipulations [54].

Additionally, the doxycycline-inducible *Kras* system (iKras) allows for temporary control of the gene expression. Collins et al. generated triple transgenic R26-rtTa-IRES-EGFP; TetO-*Kras^G12D^*; *p48*-Cre mice [55]. Upon doxycycline administration, the reverse tetracycline transactivator (rtTa)-doxycycline complex, which is specifically expressed in the pancreas controlled by p48-Cre, binds to the tetracycline-responsive element (TRE), allowing downstream *Kras^G12D^* expression. They found that inactivation of *Kras^G12D^* in established precursor lesions resulted in regression of these lesions, demonstrating that sustained *Kras^G12D^* expression is essential for tumor maintenance.

The avian sarcoma-leukosis virus-A-derived vector, namely RCAS (replication-competent avian sarcoma-leukosis virus long terminal repeat with splice acceptor), can deliver cDNA, shRNAs, non-coding RNAs, or CRISPR components to tumor virus A (TVA)-expressing cells under the control of promoters like elastase [56]. The RCAS-TVA system allows for versatile gene delivery in transgenic mice expressing the TVA receptor. In *LSL-Kras^G12D^*; *Ptf1a-cre*; *elastase-tva* mice, injected DF1 chicken fibroblasts producing RCAS-*Wnt1*, -*GFP*, or -β-*catenin^S37A^* selectively control Wnt signaling in pancreatic tissues already primed for oncogenic *Kras* expression [57]. This approach provides the flexibility to combine multiple genetic alterations for investigating various molecular mechanisms.

CRISPR-based somatic genome engineering has emerged as a powerful method for modeling PDAC with the advantage of increased speed, flexibility, and cost-effectiveness. Two main delivery methods have been developed for in vivo CRISPR editing in PDAC models: viral and non-viral approaches [58]. Viral delivery systems, particularly adeno-associated viruses (AAVs), have been widely adopted. In one common approach, *Kras^LSL-G12D^*; *Rosa26^CAG-LSL-Cas9^*; *Ptf1a^Cre^* mice express oncogenic *Kras*^G12D^ and Cas9 specifically in pancreatic cells. Researchers can then deliver vectors encoding sgRNAs by using self-complementary AAV targeting genes of interest, such as *Trp53*, via retrograde pancreatic ductal injection. Non-viral delivery methods, such as pancreas electroporation, offer an alternative approach with potentially reduced immunogenicity and larger cargo capacity [58]. In this technique, plasmid DNA encoding *Cas9* and sgRNAs is directly introduced into pancreatic cells through electrical pulses. The choice of plasmids often depends on the specific experimental design. In mice that already express Cas9 (e.g., *Rosa26^CAG-LSL-Cas9^* strains), only sgRNAs need to be delivered. Conversely, in wild-type mice, vectors containing both *Cas9* and sgRNAs should be used. These CRISPR-based approaches have significantly accelerated PDAC research, allowing for rapid screening of potential tumor suppressors and oncogenes. The in vivo CRISPR screening method will be further reviewed in the following sections.

## 4. CRISPR-Cas9 Screening Models

**Figure 3:**
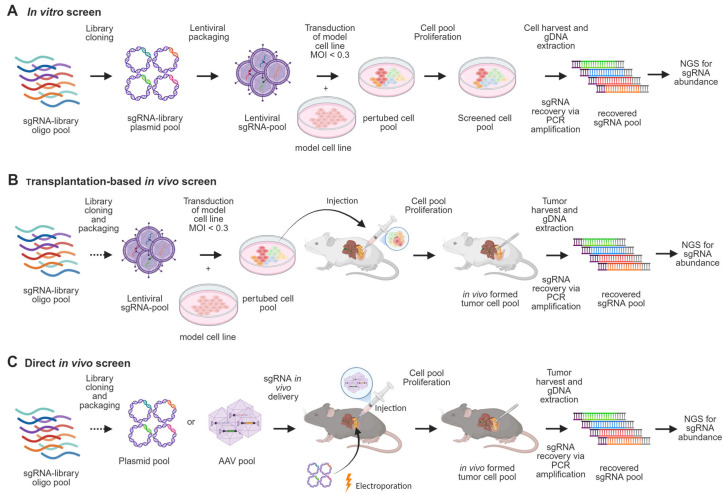
Screening methods in PDAC research. (**A**) CRISPR in vitro screening begins with cloning an sgRNA pool into a library, which is used to produce a lentiviral pool. The model cell line is transduced at a multiplicity of infection (MOI) < 0.3 to ensure only one sgRNA integrates per cell. The perturbed cell pool is then expanded for the required number of doublings, followed by genomic DNA extraction and PCR amplification of sgRNA sequences to determine their abundance. (**B**) For in vivo screens using transplantation-based methods, the perturbed cell pool is injected intravenously, subcutaneously, or orthotopically into the host model. (**C**) Alternatively, somatic in vivo screening methods deliver sgRNA libraries directly into pancreatic tissue through electroporation or AAV injection. In both cases, tumor formation is monitored, and sgRNA abundance is analyzed post-harvest.

CRISPR-Cas9-based genetic screening is a powerful tool for high-throughput functional genomics. Typically, sgRNA libraries are delivered via lentiviral vectors alongside Cas9 to induce gene knockouts in cell populations, with proliferation-based drop-out assays used to identify fitness or drug-sensitivity genes (Figure 3A) [59,60,61]. While informative, such systems lack the complexity of in vivo tumor biology. To address this limitation, CRISPR screening has been adapted to in vivo models, where transplantation-based approaches introduce perturbed cell pools derived from xenografts or established lines into host animals, offering a level of robustness comparable to in vitro systems [62,63,64] (Figure 3B). This enables assessment of gene function under physiological conditions and has revealed both conserved and context-specific metabolic dependencies. For example, in vivo screens, compared with in vitro screens, have identified genes regulating heme metabolism, oxidative phosphorylation, nucleotide synthesis, and antigen presentation as crucial for tumor formation [65]. Additionally, immune profiling using immunocompetent versus immunodeficient mice identified interferon-γ (IFNγ) as a key mediator of immune evasion and tumor suppression in pancreatic cancer [66]. Drug-assisted in vivo CRISPR screens using this platform have also uncovered targets that enhance the efficacy of gemcitabine or MEK inhibition, highlighting its therapeutic relevance [67].

More recently, somatic CRISPR screening using GEMMs has enabled direct analysis of gene function during tumor initiation (Figure 3C). In these models, sgRNA libraries are delivered via electroporation or AAV into pancreatic tissue, allowing in situ gene editing within a native microenvironment [68]. For instance, a recent study used an in vivo screening method with an adeno-associated virus-sgRNA library targeting 125 recurrently mutated PDAC genes [69]. Using KC mice with inducible Kras and Cas9-GFP, the study identified accelerated tumor formation upon Cas9-mediated knockout of suppressor genes such as *Cdkn2a*, *Rnf43*, and *Fbxw7*, validating the somatic screen’s effectiveness in phenotyping relevant PanIN development hits. Notably, novel tumor progression regulators, SCAF1 and USP15, were discovered, shown to be suppressive in pancreatic tumorigenesis. These findings significantly contribute to understanding early PDAC carcinogenesis mechanisms, providing more insights into key regulators and pathways like TGF-β regulation, MAPK signaling, and NF-κB signaling. Although this method is pivotal for addressing tumor suppressors in early PDAC development, the library size was limited due to constraints such as viral titer and transduction efficiency. To ensure each cell received only one sgRNA and to maintain statistical robustness, infection efficiencies were optimized at low levels, which inherently restricted the number of sgRNAs that could be screened.

Emerging systems like organoids, either directly cultured from patient tissue such as IPMNs [70] or derived from induced pluripotent stem cells (iPSCs) [71], offer advantages over traditional 2D cell culture by better mimicking cancer heterogeneity, making them clinically relevant for transplantation-based screening methods. However, challenges such as sgRNA efficiency, clonal heterogeneity [72], and variability in transduction efficiency and proliferation among clones [73] lead to high false-positive rates. While such approaches hold promise for functional studies, they are not yet established for pancreatic or iPSC-derived organoids. Furthermore, most in vivo screens rely on CRISPR/Cas9 or RNAi, but the rapidly expanding CRISPR toolbox offers even greater potential. Emerging technologies such as CRISPR interference (CRISPRi), CRISPR activation (CRISPRa) [74], and prime editing [75], which allows precise base conversion without double-strand breaks, enable refined modulation of gene function. Additionally, CRISPR base editing has been applied in PDAC models to correct oncogenic *KRAS* mutations in organoids and cell lines, offering a potential tool for early intervention [76]. TALENs (transcription activator-like effector nucleases), though less widely used in PDAC research, remain a valuable alternative for precise gene editing, particularly when off-target minimization is critical [77]. Alternative CRISPR systems such as Cas12 [78] and Cas13 [79] also hold promise for refined gene regulation studies in vivo. Additionally, applying CRISPR screening to in vivo models that incorporate risk factors such as pancreatitis, obesity, or diabetes as discussed in the flowing sections could provide valuable insights into early PDAC carcinogenesis.

## 5. Risk Factor: Pancreatitis

Pancreatitis is a well-established risk factor for pancreatic cancer [80,81]. While evidence regarding acute pancreatitis (AP) as a risk factor remains inconsistent [8,82], numerous studies have shown that chronic pancreatitis (CP) is particularly significant in increasing the risk of PDAC [83,84,85]. CP is a complex disease influenced by factors such as smoking [86], alcohol abuse [87], pancreatic duct obstruction [88], autoimmunity [89], and genetic mutations [90,91,92]. Hereditary CP typically begins with recurrent episodes of AP that progress to CP over 10–15 years, significantly increasing the risk of PDAC in affected individuals by creating a chronic inflammatory microenvironment. This hereditary form, often caused by mutations in genes encoding protease serine 1 (PRSS1), chymotrypsin C (CTRC), serine protease inhibitor Kazal type 1 (SPINK1), and/or carboxypeptidase A1 (CPA1), serves as a genetic background for studying the mechanisms underlying pancreatitis and its progression to PDAC.

The p.R122H mutation in the *PRSS1* gene that encodes the human cationic trypsinogen is the first reported mutation that was identified in hereditary CP [90]. In the subsequent years, several mutations like the p.N21I [93] and p.A16V [94,95] in the *PRSS1* gene were also linked to hereditary CP. All these mutations lead to elevated levels of intrapancreatic trypsin that causes damage to the organ. Trypsin is produced and secreted by the pancreas in an inactive form called trypsinogen. Trypsinogen is converted to the active form by proteolysis of its activation peptide. This proteolysis occurs by enterokinase or trypsin itself. However, mutations in trypsinogen or the effector proteins of the protective mechanisms can disturb the homeostasis and lead to pancreatitis. Several studies also reported that increased autoactivation of trypsinogen is the underlying mechanism of action for other cationic trypsinogen mutations [96,97]. The first transgenic mouse model expressing the PRSS1 mutant p.R122H was generated by Archer et al., mimicking key features of human pancreatitis, including acinar cell damage, fibrosis, and inflammation [98]. However, the penetrance of the p.R122H mutation in this model was only 40%, compared to 80% in hereditary pancreatitis patients. Another model system, developed by Athwal et al. [99], expressed human PRSS1 wild-type protein or mutants (p.R122H and p.N29I), predisposing mice to pancreatitis. Upon stimulation with low-dose cerulein, these mice progressed to PDAC. Additionally, mutations that increase trypsinogen autoactivation, such as p.D23A [100] and p.D22N/K24R [101], have been used to generate mouse models that replicate hallmarks of pancreatitis, including inflammatory cell infiltration and acinar cell necrosis. These preclinical models provide valuable tools for studying pancreatitis and early steps of PDAC.

In humans, mutations in the *CTRC* gene are strongly associated with chronic pancreatitis [102,103]. Under normal physiological conditions, CTRC protects against pancreatitis by degrading intrapancreatic trypsinogen. Loss-of-function mutations in CTRC impair this protective mechanism, increasing susceptibility to pancreatitis. Interestingly, C57BL/6N mice naturally lack CTRC expression, with chymotrypsin B1 (CTRB1) serving as the major chymotrypsin isoform in these mice. Using CRISPR-Cas9 technology, Jancsó et al. [104] generated a *Ctrb1*-deficient mouse model, which exhibited more severe cerulein-induced pancreatitis compared to controls due to elevated intrapancreatic trypsin activation. These findings highlight the critical role of chymotrypsin in maintaining protease homeostasis in the pancreas.

Another defense strategy of the pancreas against trypsinogen autoactivation is the enzyme SPINK1, which inactivates prematurely activated trypsin and protects against pancreatitis [105]. Over 20 SPINK1 variants have been reported, with the p.N34S mutation being the most frequent [91]. Given SPINK1’s critical role in the human pancreas, several mouse models have been developed to elucidate its mechanisms and explore its potential as a therapeutic target. It is important to note that the mouse ortholog of human SPINK1 shares 63% amino acid sequence identity with the human protein, while rats possess homologous proteins known as pancreatic secretory trypsin inhibitors I and II (PSTI-I and -II) [106]. While *Spink1*-null mice (previously referred to as Spink3-null) did not survive beyond two weeks [107], heterozygous *Spink1*-deficient mice exhibited reduced SPINK1 expression without altered susceptibility to transient cerulein-induced pancreatitis. However, recent studies demonstrated that prolonged cerulein hyperstimulation in *Spink1-KO^het^* mice led to hallmarks of acute pancreatitis, underscoring SPINK1’s protective role in trypsin-dependent pancreatitis [108]. Furthermore, when crossed with trypsinogen mutant strains (T7D23A or T7D22N/K24R), which spontaneously develop CP, *Spink1-KO^het^* mice exhibited accelerated CP progression, suggesting that even slightly reduced SPINK1 levels can exacerbate CP development [108]. These findings underscore SPINK1’s significance as a disease modifier in pancreatitis and its potential relevance to therapeutic strategies.

Recent studies have shown that CP can also occur through mechanisms independent of trypsin-dependent pathways. The second most abundant enzyme in pancreatic juice, pro-carboxypeptidase A1 (proCPA1) [109], is activated to its functional form, CPA1, by trypsin and CTRC [110]. DNA sequencing of 944 individuals with CP showed that loss-of-function CPA1 variants play a role in the development of CP. Forty-one percent of individuals with functionally impaired CPA1 variants carried the c.768C > G (p.Asn256Lys) mutation [92]. In 2019, a mouse model that recapitulated CPA1-associated CP was developed [111]. Experiments using this model demonstrated that misfolding of the mutant CPA1 protein induces endoplasmic reticulum (ER) stress, leading to CP. Importantly, this model differs from trypsin-dependent models by highlighting ER stress as a distinct mechanism in CP pathogenesis, underscoring the complexity of the disease and the need to investigate multiple molecular pathways.

Cerulein, a cholecystokinin analogue, is the most commonly used agent to induce acute or chronic pancreatitis in rodents. The severity of pancreatic damage in cerulein-induced models is directly proportional to the dose and duration of administration, making it a reproducible and widely used experimental system. Studies by Guerra et al. indicate that activation of oncogenic *Kras^G12V^* in adult acinar cells alone does not lead to PDAC; however, pretreatment with cerulein to induce CP results in PDAC development, suggesting that cerulein sensitizes the pancreas for tumorigenesis [112]. Nonetheless, a major drawback of cerulein-induced mouse models is that they do not represent the clinical situation, where various factors such as alcohol abuse, gallstones, or genetic predisposition contribute to pancreatitis.

Several important factors must be considered when selecting a mouse model to study the role of CP in PDAC. The model should faithfully replicate the stages of human disease and allow disease induction with minimal artificial intervention. For instance, mouse models that develop pancreatitis while harboring genetic predispositions to PDAC provide a more patient-relevant platform. It is also important to note that early steps of PDAC and CP share similar histopathological features such as ADM and inflammatory cell infiltration, and hence these mouse models offer the opportunity to understand the different stages of the diseases in a context-dependent manner. Furthermore, CP and PDAC develop over years in humans, involving mutation accumulation, DNA damage, and neoplastic lesion formation. Mouse models offer the advantage of recapitulating these processes under controlled conditions, enabling comprehensive studies of genetic and environmental influences on disease progression. Combining the described genetic or chemically induced pancreatitis models with well-established cancer models, such as inducible *Kras*^G12D^ mice, can enhance our understanding of CP as a risk factor for PDAC and provide insights into the molecular mechanisms driving this progression.

## 6. Risk Factor: Obesity

Obesity is a chronic disease characterized by abnormal and excessive fat accumulation, posing significant health risks worldwide. Since 1990, adult obesity rates have more than doubled, while adolescent obesity has quadrupled globally, contributing to a growing public health crisis [113]. A hallmark of obesity is hypertrophic and dysfunctional adipose tissue, which acts not only as an energy storage depot but also as an endocrine and immunologically active organ [114]. By releasing adipokines, it influences both local microenvironments and distant organs systemically [115]. Consequently, obesity is associated with systemic and chronic low-grade inflammation [116].

**Figure 4:**
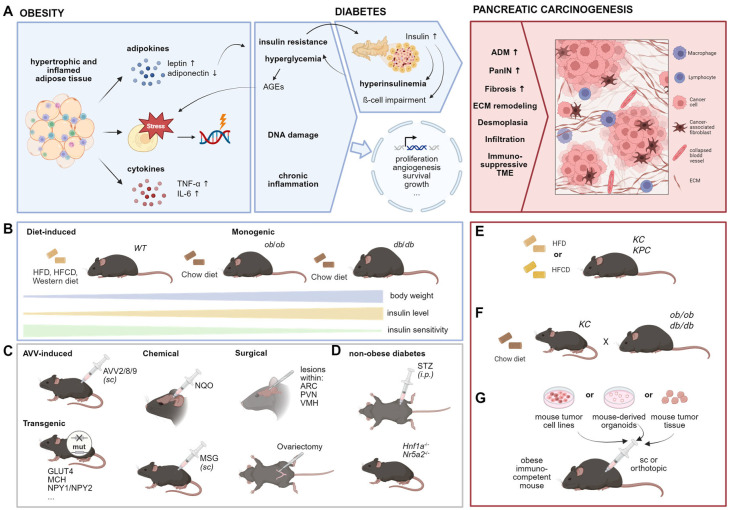
Animal models to investigate the impact of obesity on PDAC. (**A**) Selected hypothesized links between obesity and early pancreatic carcinogenesis. Dysfunctional adipose tissue results in cellular stress and altered hormone and cytokine secretion causing DNA damage and a status of chronic inflammation, respectively (**left**). Obesity can further result in diabetes (**middle**) in which insulin resistance causes increased insulin production within pancreatic islets, which in turn leads to hyperinsulinaemia and β-cell impairment. The resulting hyperglycaemia promotes the generation of advanced glycation end products (AGEs) which further fuel cellular stress. The mentioned links may promote altered gene expression of processes associated with carcinogenesis, promoting features of early pancreatic carcinogenesis (**right**). ADM: acinar to ductal metaplasia, PanIN: pancreatic intraepithelial neoplasia, ECM: extracellular matrix, TME: tumor microenvironment. (**B**) Common obesity mouse models. HFD: high-fat diet, HFCD: high-fat, high-calorie diet. (**C**) Additional models to induce obesity in rodents. AVV: adeno-associated virus vector, *sc*: subcutaneous, NQO: 4-nitroquinoline 1-oxide, MSG: monosodium glutamate, ARC: arcuate nucleus, PVN: paraventricular nucleus, VMH: ventromedial hypothalamus. (**D**) Mouse models to induce non-obese diabetes. STZ: streptozotocin, *i.p.*: intraperitoneal (**E**) Combination of genetic obesity and PDAC models. (**F**) Diet-induced obesity in PDAC mouse models. (**G**) Syngeneic models to investigate tumor progression in the context of obesity.

Over recent decades, obesity has been identified as a significant risk factor for numerous chronic diseases, including cardiovascular conditions, type 2 diabetes, and various cancers such as PDAC [9,117,118]. Additionally, obesity elevates the risk of developing pancreatic precancerous lesions [119]. Preclinical studies suggest several mechanisms by which obesity promotes pancreatic tumorigenesis, including hyperinsulinaemia and insulin resistance, hyperglycaemia, inflammation, altered cellular metabolism, hormone dysregulation, cellular stress, microbial dysbiosis, as well as activation of oncogenic drivers [120,121,122] (Figure 4A).

Numerous murine obesity models have been studied in this context [123] and reviewed in detail by Kfoury et al., among others [123,124,125,126]. The most commonly used models are genetically engineered or diet-induced obesity (DIO) models (Figure 4B). For example, the *ob*/*ob* and *db*/*db* mice are monogenic models that represent mutations in the leptin gene or its receptor, respectively. These mutations lead to hyperphagia, resulting in severe early-onset obesity and, in the case of *db*/*db* mice, diabetes [127]. The *ob*/*ob* mice exhibit impaired glucose tolerance and insulin sensitivity reflecting a more pre-diabetic stage, whereas *db*/*db* mice are insulin resistant and display hyperinsulinemia as well as hyperleptinaemia. Obesity can also be modeled using other approaches, including nutritional, surgical, chemical, and AAV methods (Figure 4C), which differ from non-obese diabetes models (Figure 4D) that will be discussed in the following section.

To investigate the effect of obesity on pancreatic carcinogenesis, recent studies have combined obesity models with PDAC models. For instance, crossing *ob*/*ob* mice with KC mice generates KCO mice (Figure 4E) [128], which develop early-onset obesity and show increased pancreatic tumor burden. Chung et al. have detailed the impact of obesity on early pancreatic carcinogenesis and demonstrated that weight loss—induced either by caloric restriction or exogenous AAV-leptin administration—can reverse these effects [128]. They also identified an endocrine–exocrine signaling pathway involving cholecystokinin derived from stressed β-cells in pancreatic islets, highlighting local obesity-associated changes as pivotal in early pancreatic carcinogenesis. However, several studies have highlighted leptin as an adipokine with both systemic and obesity-independent effects, acting as a potent anti-apoptotic, proliferative, and inflammatory agent [129,130], which significantly influences the immune response [131,132] and thus may directly impact tumorigenesis. In vitro studies further showed that leptin increases the migratory capacity of pancreatic cancer cells [133,134,135]. Given these findings, models based on modifications in leptin signaling, such as *ob*/*ob* mice, may not accurately reflect the microenvironment of pre-neoplastic lesions or pancreatic cancer. Additionally, *db*/*db* mice are less suitable for studying obesity-related pancreatic carcinogenesis due to confounding effects from diabetes. Non-diabetic obesity models are therefore recommended to ensure more accurate results.

Another commonly used model to investigate obesity-related effects on pancreatic carcinogenesis is the DIO model (Figure 4F). These models involve feeding wild-type or transgenic mice high-caloric diets, resulting in a slower onset of obesity compared to *ob*/*ob* and *db*/*db* mice, which more closely mimics the human pathogenesis of obesity. Chronic high-fat diet (HFD) consumption in mice leads to glucose intolerance, impaired insulin sensitivity, and enhanced β-cell mass and proliferation [136,137,138,139], reflecting a pre-diabetic stage that progressively worsens over time. Several studies have demonstrated that HFD-induced obesity promotes pancreatic cancer development in KC and KPC mice [122,128,140,141,142,143,144]. HFD has been shown to increase inflammation, fibrosis, and PanIN lesions while accelerating progression into more aggressive PDAC [122,140,144,145]. Notably, it has been suggested that HFD contributes to pancreatic cancer not only through obesity and pre-diabetes but also by directly affecting carcinogenic processes [146]. However, the HFD of these studies varied in composition, fatty acid (FA) ratio, and duration and onset of intervention. Ead et al. reported that feeding KC mice an HFD with 60% of calories from fat (based on lard; 9:1 n-6:n-3 FA ratio) resulted in only mild effects on early pancreatic ADM without cancer development [142]. In contrast, a high-fat, high-calorie diet with 40% of calories from corn oil (50:1 n-6:n-3 FA ratio) in KC mice led to more advanced PanIN lesions [144] and tumor development already at an age of 3 months [141]. Furthermore, KPC mice fed a HFD (60% calories from fat; source: beef tallow and safflower oil) developed larger primary tumors and exhibited higher metastatic rates [143]. Compared to HFD, a high-carbohydrate diet displayed lower tumorigenic potential, while a high-protein diet was comparable to a normal diet [147]. These findings emphasize the significance of factors such as calorie percentage from fat, fat source, and n-6:n-3 FA ratio in influencing the obese phenotype and metabolic signaling linked to pancreatic carcinogenesis [121]. Additionally, when interpreting results from DIO models, researchers must consider confounding variables such as housing conditions, microbiome composition, age, gender, and feeding duration.

An alternative experimental design involves combining KC mice with gene therapy, where AAV vectors (AVV2/9/8) [140,148,149,150] are injected into the adipose tissue to induce obesity. Using this strategy, the precise timing of obesity induction based on the experimental design can be controlled, thereby avoiding undesired effects on embryonic development and hormone-related processes [126].

Since in humans, obesity develops gradually over time and pancreatic cancer-driving mutations typically occur in adulthood, GEMMs for PDAC that present mutations from birth may not accurately replicate human disease pathogenesis, especially when combining two GEMMs (e.g., KCO mice). Therefore, inducing pancreatic carcinogenesis in pre-existing obesity models by inducible oncogene mutations [151] or orthotopic implantation models—using PDAC cells, organoids [152] or tumor xeno-/allografts—offer promising tools to study the impact of obesity onset on pancreatic cancer progression (Figure 4G).

## 7. Risk Factor: Diabetes

Diabetes and pancreatic cancer share some concurrent hereditary predispositions, particularly in subgroups of patients with family history and younger age at diagnosis, and these concomitant genetic susceptibilities have been comprehensively reviewed recently by Popovic et al. [153]. Mutations in *HNF1A, PDX1, and HNF1B*, which affect pancreatic development, have been associated with increased risk of monogenic forms of maturity onset diabetes of the young (MODY types 3, 4, and 5) and pancreatic cancer [154,155]. For instance, by crossing KC mice with *Hnf1a^flox/flox^* mice, Kalisz et al. found that *Hnf1a* loss cooperates with the *Kras^G12D^* mutation to promote pancreatic carcinogenesis [156]. The nuclear receptor NR5A2 plays a dual role in pancreatic health and disease. Agonistic activation of NR5A2 promotes beta cell regeneration, potentially reversing type 1 diabetes [157]. Conversely, *Nr5a2* heterozygosity sensitizes the pancreas to pancreatitis-induced inflammation, delays the ADM regeneration, and accelerates oncogenic *Kras*-driven carcinogenesis in mice [158,159]. Additionally, *UCP2* gene polymorphisms are associated with both diabetes and PDAC, with UCP2 loss significantly reducing oncogenic *Kras*-induced PDAC growth [160]. These findings collectively indicate that certain genetic factors contribute to both diabetes and pancreatic cancer susceptibility, and designing mouse models for understanding these shared genetic pathways may provide new insights into prevention strategies and targeted therapies for both conditions.

Type 2 diabetes has been associated with an approximately two-fold overall increased risk of pancreatic cancer, with the highest risk observed in the first year following diabetes diagnosis gradually diminishing over time [161,162]. Interestingly, recent studies have revealed that type 1 diabetes patients also exhibit modestly elevated hazard ratios for pancreatic cancer, with men showing a slightly higher risk than women [163]. This similarity in risk profile between type 1 and type 2 diabetes suggests that the underlying mechanism may be related to shared metabolic pathology, likely chronic hyperglycaemia, rather than factors specific to type 2 diabetes. The strong association between hyperglycaemia and pancreatic cancer risk is further supported by the finding that each 0.56 mmol/L (10 mg/dL) increment in fasting glucose is associated with a 14% increase in the incidence rate of pancreatic cancer [164]. Experimental evidence from mouse models has provided mechanistic insights into this relationship. Non-obese-associated diabetes induced by streptozotocin accelerates the PDAC development in KC mice (Figure 4D), an effect that can be prevented by scavenger of reactive carbonyl species (RCS) and advanced glycation end-product (AGE) inhibitor, indicating that hyperglycaemia-derived carbonyl stress plays a crucial role in pancreatic carcinogenesis, particularly in high-risk diabetic individuals [165]. These findings also highlight the importance of glycaemic control and cancer surveillance in all diabetic patients, regardless of diabetes classification.

Diabetes and pancreatic cancer share some common risk factors, such as obesity—as earlier mentioned—highlighting the complexity and overlap of underlying mechanisms [166]. Obesity-induced insulin resistance is a critical link in this relationship, contributing to both diabetes and carcinogenesis [167]. Experimental evidence supports this connection, as obese-associated diabetic *db*/*db* mice demonstrate accelerated PDAC progression and metastasis [168]. To elucidate the specific role of insulin in PDAC initiation, Zhang et al. generated LSL-*Kras*^G12D^; *Ins1*^+/−^; *Ins2*^−/−^; *Ptf1a*^CreER^ mice with genetic manipulations of *Ins1* and *Ins2* resulting in a sustained reduction in fasting insulin while maintaining unimpaired glucose homeostasis in female mice. Their findings revealed that HFD-mediated increases in insulin, in the absence of changes in fasting glucose, promoted PanIN development [169]. In a case-control study involving 973 patients with PDAC, Li et al. found that insulin or insulin secretagogues increased the risk of PDAC, while metformin administration in diabetic patients lowered the risk [170]. Preclinical studies have further demonstrated that metformin suppresses PDAC initiation and progression in KC and KPC mice [171]. These findings underscore the importance of further investigating the causal relationship between pharmacological therapies for type 2 diabetes and PDAC development.

New-onset diabetes is significantly associated with an increased incidence of pancreatic cancer compared to long-term diabetes (more than 2 years) prior to pancreatic cancer diagnosis, suggesting a potential reverse causation, where diabetes occurs as a consequence of PDAC development and may present as a subclinical manifestation [172]. Supporting this hypothesis, Parajuli et al. demonstrated in a KC mouse model that TGF-β signaling-activated apoptosis during PDAC progression caused depletion of β-cell mass, resulting in diabetic susceptibility [173]. This evidence that pancreatic cancer can trigger diabetes by damaging islet β-cells further contributes to the complexity.

In summary, it is crucial to recognize that chronic inflammation, hyperglycaemia, and insulin resistance can contribute to pancreatic carcinogenesis, consistent with the observation that long-term type 2 diabetes patients have an increased pancreatic cancer incidence compared to those with short-term type 2 diabetes (2–5 years) [172]. Besides, diabetes is often associated with comorbidities such as obesity and pancreatitis, which are themselves risk factors for pancreatic cancer. This complexity underscores the importance of precisely defining mouse models to avoid confounding factors and clearly delineate specific causal relationships. Conversely, pancreatic cancer development can also cause the onset of diabetes. These bidirectional relationships highlight the importance of carefully designing relevant mouse models to decipher the underlying mechanisms.

## 8. Other Risk Factors

Other than the previously mentioned risk factors that have been extensively investigated, additional cancer risk factors such as smoking, alcohol consumption, and infections [174] are also explored in GEMMs concerning the epidemiology of pancreatic cancer.

Patients with a history of pancreatitis, especially heavy smokers, exhibit a significantly elevated risk of developing pancreatic cancer [162]. Data regarding the impact of smoking on PDAC carcinogenesis is inconsistent. For instance, KC mice exposed to cigarette smoke for two weeks experienced significant weight loss but showed decreased cell proliferation in pancreatic ductal and acinar cells [175]. However, a six-week exposure to tobacco smoke promoted the formation of PanINs in KC mice, which was further exacerbated by the induction of chronic pancreatitis [176]. This discrepancy may arise from variations in the source, duration, and concentration of cigarette smoke used in different studies. Another investigation exposed KC mice to cigarette smoke for up to 20 weeks and observed overexpression of stem cell features such as *Paf1* and *Sox9*; this stemness may contribute to the expansion of PanINs [177]. Nicotine, the primary addictive substance in cigarette smoke, has been shown to accelerate ADM and tumor formation by activating AKT/ERK/MYC signaling in both KC and KPC mice [178]. When interpreting these results, it is essential to consider confounding factors such as smoking-induced genotoxicity [179] and its interplay with other risk factors like diabetes, obesity, and chronic pancreatitis. These interactions have been extensively reviewed elsewhere [180].

Excessive alcohol consumption is associated with an increased risk of pancreatic cancer [6]. Moderate alcohol intake has been shown to lead to the development of advanced PanIN and subsequent PDAC in KC mice [181]. However, it is important to note that alcohol is also a leading cause of pancreatitis, which can act as a confounding factor in this context.

Infection with pathogens such as Hepatitis B virus and *Helicobacter pylori* has been identified as potential risk factors for PDAC, highlighting the role of microbiome alterations and dysfunction in the disease’s development. Studies have investigated the gut microbiome in KC mice [182] and the fecal and tumoral microbiome in KPC mice [183] to better understand the relationship between microbiome composition and PDAC development. Additionally, the oral microbiome, particularly *Porphyromonas gingivalis*, has been linked to an increased risk of pancreatic cancer [184], prompting further investigation using GEMMs. Administration of *Porphyromonas gingivalis* induced pancreatic ADM in wild-type mice and accelerated PDAC progression from PanIN lesions in *Kras^G12D/+^*; *Ptf1a^ER-Cre/+^* mice, while also altering the intrapancreatic microbiome composition [185]. Furthermore, ablation of the gut microbiome in *Kras^G12D/+^*; *PTEN^flox/+^*; *Pdx1-Cre* mice resulted in decreased tumor growth and a reduction in the suppressive immune microenvironment [186]. Beyond these examples, recent studies have manipulated the gut microbiome through methods such as fecal microbial transplants and systemic antibiotics or antifungals in mouse models to explore specific microbiome relationships with pancreatic carcinogenesis. This area of research has been extensively reviewed, emphasizing the potential for microbial modification as a therapeutic strategy against pancreatic cancer [187,188].

## 9. Conclusions and Perspectives

Preclinical mouse models offer diverse approaches for investigating PDAC tumor development and identifying potential therapeutic targets. While xenograft models derived from PDAC cell lines, tumor tissues, or PDOs allow for genetic manipulation and drug testing in a relatively high-throughput manner, they have limitations in monitoring early carcinogenesis and testing early triggers. Humanized models add another dimension by better mimicking the human immune system but still fall short in replicating the complete process of early tumor development. GEMMs, particularly those manipulating somatic gene expression, are invaluable for exploring genetic alterations in early pancreatic carcinogenesis within the context of a competent immune system. Although often time consuming, these models provide crucial insights into tumor initiation and progression. The new introduction of large-scale CRISPR screening has further extended the utility of GEMMs, enabling unbiased identification of key genetic players in PDAC development.

Recent research has increasingly focused on the role of inflammatory triggers in PDAC initiation. Applying environmental risk factors or targeting underlying mechanisms in GEMMs can broaden our understanding of carcinogenesis, potentially leading to early prevention strategies and the development of druggable targets to halt cancer progression at its earliest stages.

However, caution is necessary when designing and interpreting results from mouse models. While GEMMs like the KPC model display good tumor heterogeneity, they may not consistently correlate with all clinical features seen in PDAC patients, such as cachexia. Off-target effects should also be considered, particularly when using Cre-lox systems. For instance, drivers like PDX-1 and Ptf1a can be expressed in organs other than the pancreas during embryonic development. Notably, the development of thymic tumors in KC mice has been reported and should be accounted for to avoid misinterpretation of results [189]. Furthermore, inflammatory factors are often interconnected and can act as confounders for each other, necessitating the careful interpretation of results. Finally, it is crucial to verify the translational relevance of findings from mouse models in human systems before drawing conclusions.
